# Association Between Maternal Bladder Descent Angle and Urinary Incontinence in Late Pregnancy: A Transperineal Ultrasonography Study

**DOI:** 10.1111/luts.70050

**Published:** 2026-02-03

**Authors:** Ryoko Minami, Yumiko Tateoka, Akihiro Kawauchi

**Affiliations:** ^1^ Doctoral Program (Nursing Science) Shiga University of Medical Science Otsu Shiga Japan; ^2^ Division of Practice Nursing Shiga University of Medical Science Otsu Shiga Japan; ^3^ Department of Urology Otsu City Hospital Shiga, Otsu Japan

**Keywords:** bladder descent angle, bladder morphology, pregnancy, transperineal ultrasonography, urinary incontinence

## Abstract

**Objective:**

To quantitatively evaluate bladder morphological changes induced by fetal head descent during late pregnancy using transperineal ultrasonography (US) and to investigate the association with urinary incontinence (UI). This study aimed to introduce a novel, imaging‐based approach for assessing pregnancy‐related urinary dysfunction.

**Methods:**

In this study, 14 women with singleton pregnancies beyond 36 weeks of gestation were evaluated. During routine antenatal visits, participants completed a validated questionnaire assessing urinary symptoms, and the bladder descent angle (BDA) was measured using transperineal US. The BDA was defined as the angle between the bladder base and the inferior margin of the pubic symphysis.

**Results:**

UI was reported in 57.1% of participants, with stress urinary incontinence (SUI) being the most common subtype (62.5%). Ultrasonography revealed that BDA increased in late pregnancy. The SUI group exhibited a significantly greater BDA compared with the no‐UI group (*p* = 0.03), whereas the overall UI group showed only a non‐significant trend (*p* = 0.081).

**Conclusions:**

The BDA assessed by transperineal US is considered a practical and simple marker for the evaluation of UI.

## 1 Introduction

Urinary incontinence (UI) is a common condition associated with pregnancy and childbirth, negatively affecting the quality of life of women worldwide. The prevalence of UI reportedly increases from 8.9% before pregnancy to 39.8% during pregnancy and remains elevated postpartum [1]. Numerous studies have identified various risk factors, including age, parity, body mass index (BMI), gestational weight gain, menopausal status, previous history of UI, vaginal birth, uterine fundoplication, episiotomy, pre‐pregnancy weight, smoking, constipation, infant birth weight, gestational age at delivery, and instrumental delivery [2, 3, 4, 5, 6]. These findings suggest that both pregnancy‐related hormonal changes and mechanical stress on the pelvic floor contribute to the pathophysiology of UI.

Despite extensive research, the mechanical mechanisms linking fetal head descent to lower urinary tract dysfunction remain incompletely understood. During late pregnancy, uterine enlargement and fetal head descent may exert direct pressure on the bladder and urethra, altering bladder geometry and urethral support. Such morphological changes could reduce urethral closure pressure and impair the transmission of intra‐abdominal pressure, thereby predisposing women to stress urinary incontinence (SUI). However, most previous studies have relied on subjective assessment or indirect measurements rather than imaging‐based quantification of structural changes.

Transperineal ultrasonography (US) has recently emerged as a valuable, noninvasive imaging technique for visualizing pelvic anatomy during labor. The International Society of Ultrasound in Obstetrics and Gynaecology (ISUOG) issued guidelines in 2018 emphasizing its utility in assessing fetal head descent and rotation in real time [7]. Compared with traditional vaginal examinations, transperineal US provides an objective and reproducible evaluation of pelvic structures, and its clinical application has rapidly expanded in obstetrics practice.

Building upon these advances, the present study focused on the quantitative evaluation of bladder descent caused by fetal head descent during late pregnancy. Preliminary observations using transperineal US suggested that the bladder becomes flattened and elongated compared with its nonpregnant form, which has not been previously described in the literature. Inspired by the angle of progression (AoP) used to evaluate fetal head descent, we introduced a novel parameter, the bladder descent angle (BDA), defined as the angle between the bladder base and the inferior border of the pubic bone. Although a comprehensive three‐dimensional assessment of bladder shape (e.g., volume, curvature, or cross‐sectional contours) would be ideal, this simple and reproducible two‐dimensional metric derived from sagittal imaging was feasible in the current clinical setting.

Therefore, this study aimed to (1) quantitatively assess bladder morphological changes during late pregnancy using the BDA, and (2) examine the association between BDA and UI. By introducing an objective, imaging‐based parameter, this study sought to clarify the mechanical pathophysiology of pregnancy‐related UI and establish a foundation for future longitudinal and large‐scale investigations.

## 2 Materials and Methods

### 2.1 Participants

This study included 14 women with singleton pregnancies who visited the study facility after 36 weeks of gestation between June 2022 and July 2023. The eligibility and exclusion criteria were as follows.

#### 2.1.1 Eligibility Criteria

1. Age 20–45 years.
2. Planned vaginal delivery.
3. Provision of written informed consent.

#### 2.1.2 Exclusion Criteria

1. Scheduled cesarean section.
2. Renal or urological disorders.
3. History of pelvic organ prolapse or pelvic surgery.
4. Complicated psychiatric disorders poorly controlled by medication.

### 2.2 Data Collection

#### 2.2.1 Basic Participant Information

Data on maternal age, height, weight, estimated fetal weight, biparietal diameter (BPD), fetal position, time since last urination, gestational age, obstetric history, and degree of fetal head descent (De Lee's station) were obtained from medical records. Fetal position was classified as left Occiput (LO) when the fetal back was on the left side of the mother's body and right occiput (RO) when on the right.

#### 2.2.2 Questionnaire Survey

Subjective urinary symptoms were assessed using the International Consultation on Incontinence Questionnaire–Short Form (ICIQ‐SF). The validated Japanese version was used in this study, which demonstrated a Cronbach's alpha of 0.78, indicating acceptable reliability [8].

#### 2.2.3 Measurement of the Bladder Descent Angle (BDA)

**FIGURE 1:**
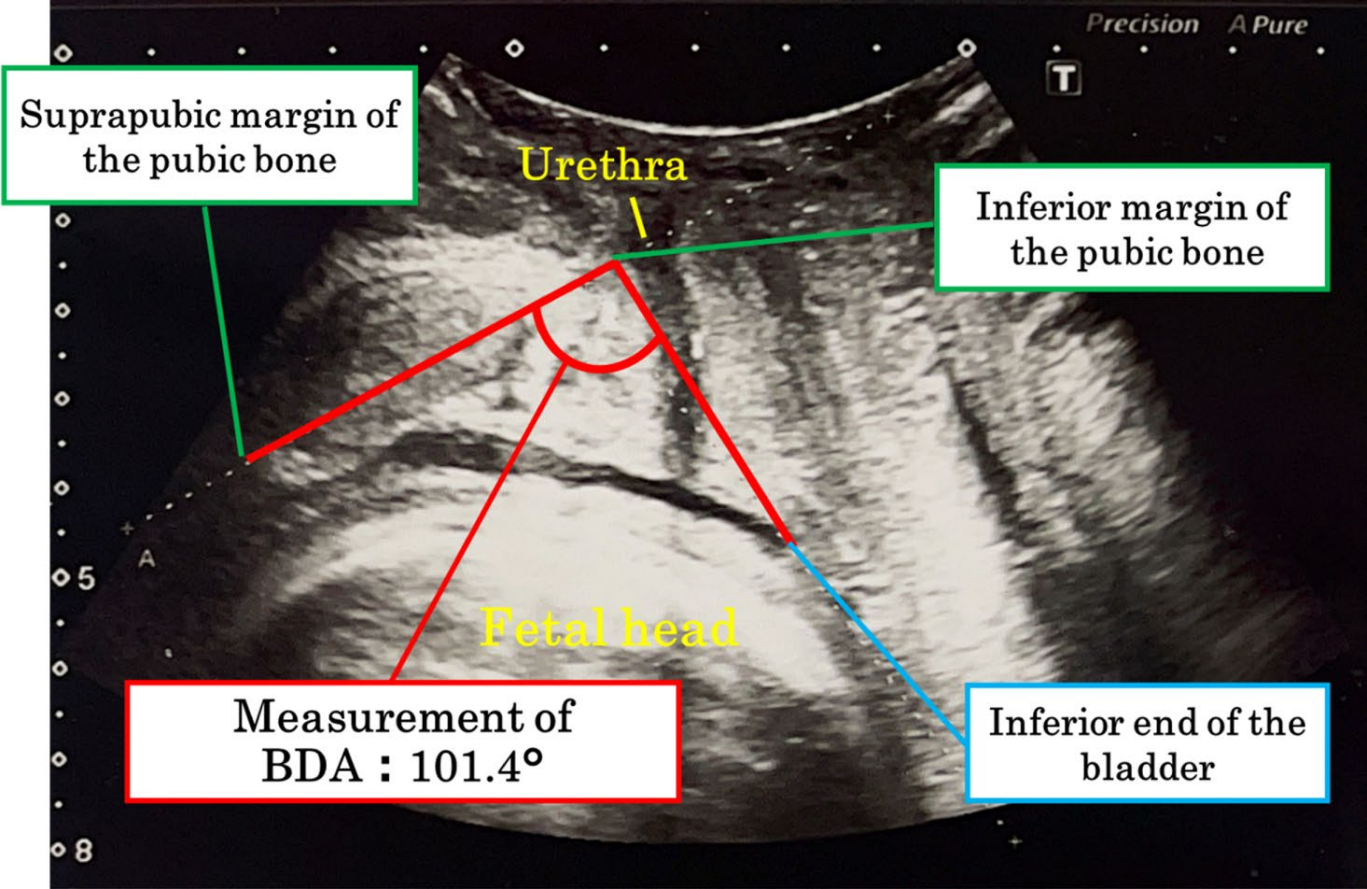
Measurement of the bladder descent angle (BDA). The BDA was defined as the angle between the long axis of the pubic bone and a line drawn from the inferior margin of the pubic bone to the inferior end of the bladder. This two‐dimensional ultrasonographic measurement provides a simple, reproducible, and objective marker of bladder displacement during late pregnancy.

Transperineal ultrasonography (US) was performed to assess the bladder descent angle (BDA) (Figure 1). To standardize bladder filling, images were obtained 60–90 min after the last voiding. Participants were positioned in the lithotomy position on the same examination table. Ultrasound gel and a sterile probe cover were applied, and the probe was positioned longitudinally along the labia to obtain a sagittal image including the pubic bone, bladder, urethra, cervix, and vagina. The long axis of the pubic bone served as an anatomical landmark.

##### 2.2.3.1 Definition of BDA

The BDA was defined as the angle between (1) the long axis of the pubic bone and (2) a line drawn from the inferior margin of the pubic bone to the lowest point of the bladder (Figure 1).

##### 2.2.3.2 Reliability Analysis

To confirm measurement reproducibility, two examiners (A and B) independently evaluated the same US images. Each examiner performed three repeated measurements on 14 participants to assess intra‐rater reliability, followed by inter‐rater reliability testing based on the first measurement from each examiner.

##### 2.2.3.3 Intra‐Rater Reliability

Examiner A: Single‐measure ICC (1,1) = 0.986 (95% CI: 0.967–0.995), Average‐measure ICC (1,3) = 0.995 (95% CI: 0.989–0.998, *p* < 0.001).

Examiner B: Single‐measure ICC (1,1) = 0.984 (95% CI: 0.963–0.994), Average‐measure ICC (1,3) = 0.995 (95% CI: 0.987–0.998, *p* < 0.001).

Both examiners demonstrated excellent intra‐rater reliability. The mean BDA values were 110.85° (Examiner A) and 110.45° (Examiner B), and this difference was clinically negligible.

##### 2.2.3.4 Inter‐Rater Reliability

A two‐way random‐effects model with absolute agreement [ICC (2,1)] was used. The single‐measure ICC was 0.328 (95% CI: −0.262 to 0.726, *p* = 0.128), and the average‐measure ICC (2, k) was 0.494 (95% CI: −0.710 to 0.841, *p* = 0.128), indicating poor inter‐rater reliability. This variation may reflect minor differences in probe placement or in identifying the lowest point of the bladder between examiners. Therefore, measurements from Examiner A were used for all subsequent analyses.

### 2.3 Statistical Analysis

All statistical analyses were performed using IBM SPSS Statistics for Windows, version 29 (IBM Corp., Armonk, NY, USA). Continuous variables are presented as mean ± standard deviation (SD) if normally distributed, and as median (interquartile range) otherwise. Group comparisons were conducted using unpaired *t*‐tests or Mann–Whitney U tests for continuous variables and Pearson's chi‐square tests for categorical variables, with a significance threshold of *p* < 0.05.

To explore the association between the presence of urinary incontinence (UI) during pregnancy and potential predictors, an exploratory logistic regression analysis was conducted. Given the small sample size (*n* = 14), bootstrap resampling (1000 iterations) was performed to assess the stability of model estimates. Model fit was evaluated using the Hosmer–Lemeshow goodness‐of‐fit test.

### 2.4 Ethical Approval

The study protocol was approved by the Ethics Committee of Shiga University of Medical Science (approval no. R2021‐151; approval date: December 3, 2021). All participants provided written informed consent prior to enrollment.

## 3 Results

In the ICIQ‐SF questionnaire, 2 participants answered, “approximately once a week or less,” 2 answered “two to three times a week,” 1 answered “approximately once a day,” and 3 answered “a few times a day,” indicating a total of 8 (57.1%) participants with urinary incontinence (UI). These 8 women were classified as the UI group, while the remaining 6 (42.9%) participants were classified as the no‐UI group.

Among the 8 participants in the UI group, 5 (62.5%) had stress urinary incontinence (SUI), 2 (25.0%) had mixed UI, and 1 (12.5%) had urge UI. For further analysis, the 5 participants with SUI were defined as the SUI group, a subset of the UI group.

Table 1 presents the participant characteristics of the no‐UI, UI, and SUI groups. The frequency of constipation was higher in the UI group than in the no‐UI group (Pearson's chi‐square test; *p* = 0.028), while other variables, including age, stature, weight, BMI, fetal weight, biparietal diameter (BPD), obstetric history, and fetal position, showed no significant differences between groups (all *p* > 0.05).

**TABLE 1:** Participant background characteristics.

	A: No‐UI group (*n* = 6) mean ± SD	B: UI group (*n* = 8) mean ± SD	C: SUI group (*n* = 5) mean ± SD	*p* A vs. B	*p* A vs. C
Age (years)	32.5 ± 8.4	35.3 ± 8.4	33.3 ± 8.1	N.S.	N.S.
Stature (cm)	155.8 ± 4.8	159.1 ± 2.9	159.4 ± 3.2	N.S.	N.S.
Weight (kg)	63.7 ± 8.4	68.5 ± 9.9	62.3 ± 6.1	N.S.	N.S.
BMI (kg/m^2^)	26.3 ± 4.0	27.1 ± 4.3	24.5 ± 2.1	N.S.	N.S.
Fetal weight (g)	2604.0 ± 237.0	2666.9 ± 161.4	2566.3 ± 62.7	N.S.	N.S.
BPD (mm)	89.8 ± 3.3	90.4 ± 2.6	89.4 ± 3.2	N.S.	N.S.
First childbirth *n* (%)	3 (37.5)	5 (62.5)	5 (100)	N.S.	N.S.
Having previously born children *n* (%)	3 (50.0)	3 (50.0)	0 (0)	N.S.	N.S.
Constipation *n* (%)	0 (0)	5 (62.5)	3 (60.0)	**0.028** *	N.S.
Smoking *n* (%)	2 (33.3)	4 (50.0)	2 (40.0)	N.S.	N.S.
History of urinary incontinence *n* (%)	1 (16.7)	1 (12.5)	0 (0)	N.S.	N.S.
Left Occiput (LO) *n* (%)	2 (33.3)	4 (50.0)	2 (40.0)	N.S.	N.S.
Right Occiput (RO) *n* (%)	4 (66.7)	4 (50.0)	3 (60.0)	N.S.	N.S.
				* *p* < 0.05	

*Note:* Unpaired *t*‐tests, Pearson's chi‐square tests were used.

Abbreviations: BMI, body mass index; BPD, biparietal diameter; N.S., not significant; SD, standard deviation.

* Stress incontinence group is a subset of the incontinence group.

**FIGURE 2:**
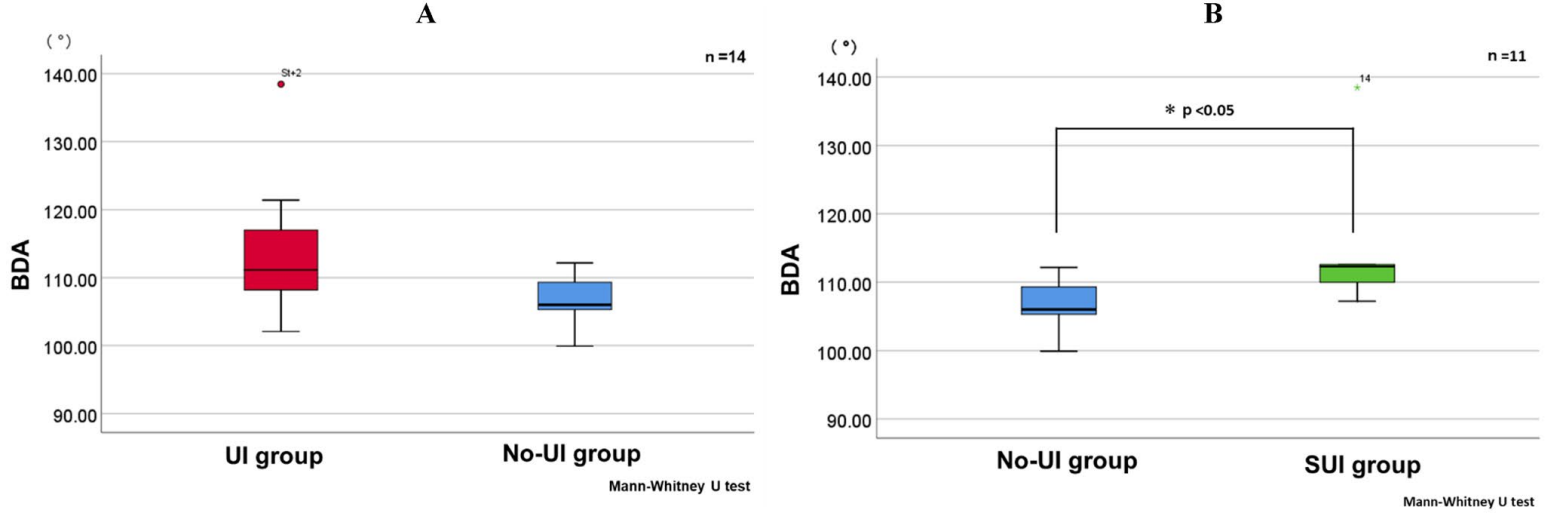
Comparison of BDA between continence groups. (A) Comparison of BDA between the UI group and the no‐UI group. (B) Comparison of BDA between the SUI group, a subset of the UI group, and the no‐UI group. A greater inferior displacement of the bladder was observed in the UI and SUI groups compared with the no‐UI group. The Mann–Whitney U test was used for between‐group comparisons.

The bladder descent angle (BDA) was 106.0° (105.3–109.3) in the no‐UI group and 111.1° (108.2–117.0) in the UI group, showing a non‐significant trend toward a higher BDA in the UI group (Mann–Whitney U test; *p* = 0.081; Figure 2A). When focusing on SUI, the BDA in the no‐UI and SUI groups was 106.0° (105.3–109.3) and 112.3° (110.0–112.6), respectively, showing a statistically significant difference between groups (Mann–Whitney U test; *p* = 0.03; Figure 2B).

A univariable logistic regression analysis demonstrated that constipation showed a trend toward association with UI (*p* = 0.065), whereas obstetric history did not (*p* = 0.304). Subsequently, an exploratory multivariable logistic regression analysis was performed including average BDA, constipation, and obstetric history to evaluate their combined effects on UI. Although the overall model reached statistical significance (Omnibus *χ* ^2^ = 9.569, df = 3, *p* = 0.023), the estimates for constipation and the intercept did not converge, indicating quasi‐complete separation of data. As a result, coefficient estimates were unstable, and reliable effect sizes could not be obtained.

To address this instability, a reduced model including only BDA and obstetric history was additionally examined. This reduced model was statistically significant (Omnibus *χ* ^2^ = 6.077, df = 2, *p* = 0.048), with Cox–Snell *R* ^2^ = 0.262 and Nagelkerke *R* ^2^ = 0.361. Average BDA showed a positive trend toward association with UI (*B* = 0.175, Wald *χ* ^2^ = 2.933, *p* = 0.087, Exp(B) = 1.192, 95% CI: 0.975–1.457), whereas obstetric history was not significantly associated (*p* = 0.452).

Bootstrapped estimates (1000 resamples) for both the full model (including BDA, constipation, and obstetric history) and the reduced model (including BDA and obstetric history) showed substantial variability in the coefficient estimates, reflecting instability due to the small sample size and quasi‐complete separation. Accordingly, all multivariable logistic regression results should be interpreted as preliminary and with caution.

## 4 Discussion

To the best of our knowledge, this is among the first studies to quantitatively evaluate maternal bladder displacement during late pregnancy using transperineal ultrasonography (US). The present findings demonstrate the relationship between bladder descent, as measured by the bladder descent angle (BDA), and subjective urinary incontinence (UI).

In this study, the prevalence of UI among women with singleton pregnancies in late pregnancy was 57.1%. Brown et al. reported prevalence rates of 10.8% before pregnancy, 17.1% in the second trimester, and 55.9% in the final trimester [9]. The prevalence observed in our study is therefore consistent with previous findings, indicating that more than half of women in late pregnancy experienced UI.

Based on the ICIQ‐SF classification, 62.5% of participants in the UI group had stress incontinence (SUI), followed by 25% with mixed UI. Thus, SUI appears to be the most common subtype of UI during late pregnancy. SUI occurs when intravesical pressure exceeds urethral closure pressure owing to transient increases in abdominal pressure during coughing, sneezing, or physical activity, resulting in urine leakage without bladder contraction [10]. Factors that reduce urethral closure pressure include urethral hypermobility and intrinsic sphincter deficiency. During late pregnancy, descent of the fetal head may further lower the urethra within the vaginal canal, thereby increasing urethral hypermobility and exerting additional stress on the bladder neck and proximal urethra [11, 12]. In cases of intrinsic sphincter deficiency, the bladder neck and proximal urethra remain partially open even at rest, so even small increases in intravesical pressure can result in urinary leakage [13, 14].

Previous studies have highlighted a close association between constipation and UI [15, 16, 17]. Rectal distension due to constipation can alter the anatomical relationship between the rectum and bladder, increase intravesical pressure, reduce bladder capacity, and trigger SUI. Furthermore, chronic constipation may impose prolonged strain on pelvic floor muscles, thereby weakening urethral support and exacerbating UI.

Using transperineal US, we observed that the bladder during late pregnancy was displaced downward beyond the inferior border of the pubic bone, with the lower portion appearing thinned and flattened, indicating morphological descent. The BDA was proportional to the extent of downward displacement, with an average BDA of 110.8° among women with singleton pregnancies during late gestation. Prior studies have primarily described anterior upward displacement associated with uterine enlargement [17], whereas our study provides novel evidence of downward bladder displacement related to fetal head descent [18].

Participants in the UI group tended to exhibit greater bladder descent than those in the no‐UI group, and a statistically significant difference was observed between the no‐UI and SUI groups. This suggests that increased bladder descent may contribute to SUI via mechanisms described in Green's classification [19].

Although exploratory logistic regression analysis including obstetric history, constipation, and BDA was performed, the primary aim was to explore the association between BDA and UI rather than to develop a predictive model. The results suggested a trend toward greater bladder descent in participants with UI; however, coefficient estimates were unstable due to the small sample size and quasi‐complete separation. Therefore, these findings should be interpreted as preliminary and hypothesis‐generating rather than confirmatory. Future studies with larger cohorts and longitudinal follow‐up are warranted to validate these associations and investigate clinical implications.

Although a comprehensive three‐dimensional evaluation of bladder shape was not feasible in the present clinical setting, the BDA offers a simple, reproducible, and objective parameter for assessing bladder displacement during late pregnancy. This metric may serve as a useful tool for identifying women at risk of SUI, with potential applications in both clinical diagnosis and grading of symptom severity.

## 5 Limitations

This study has several limitations. The small sample size (*n* = 14), partly due to COVID‐19 restrictions, limits the statistical power and generalizability of the findings. Only the two‐dimensional bladder descent angle (BDA) was assessed, without volumetric or curvature analysis. Future studies should include larger cohorts and more comprehensive assessments, such as three‐dimensional evaluation of bladder morphology and differentiation of UI subtypes.

## 6 Conclusion

The bladder descent angle (BDA) by transperineal ultrasonography (US) is thought to be a practical and simple marker for evaluation of urinary incontinence (UI). Larger, longitudinal studies incorporating a comprehensive imaging are warranted to validate these findings and assess their clinical utility.

## Funding

This work was supported by Japan Society for the Promotion of Science (JP22K21169).

## Ethics Statement

The study protocol was approved by the Ethics Committee of Shiga University of Medical Science (approval no: R2021‐151; approval date: 2021.12.03).

## Consent

All patients provided written informed consent for participation in the study and for the publication of all images, clinical data, and other data contained in the manuscript.

## Conflicts of Interest

The authors declare no conflicts of interest.

## Data Availability

The data that support the findings of this study are available on request from the corresponding author. The data are not publicly available due to privacy or ethical restrictions.
